# Short-Term Outcomes of Single-Incision Laparoscopic Surgery for Colorectal Cancer: A Single-Center, Open-Label, Non-Inferiority, Randomized Clinical Trial

**DOI:** 10.3389/fonc.2021.762147

**Published:** 2021-10-25

**Authors:** Zijia Song, Kun Liu, You Li, Yiqing Shi, Yimei Jiang, Changgang Wang, Xianze Chen, Tao Zhang, Xiaopin Ji, Ren Zhao

**Affiliations:** Department of General Surgery, Ruijin Hospital, Shanghai Jiaotong University School of Medicine, Shanghai, China

**Keywords:** colorectal cancer, single-incision laparoscopic surgery, multiport laparoscopic surgery, short-term outcomes, randomized controlled trial

## Abstract

**Objective:**

To date, well-designed randomized controlled trials examining the safety, efficacy, and long-term outcomes of single-incision laparoscopic surgery (SILS) for colorectal cancer are scarce. The aim of the current study was to compare short-term outcomes of SILS for colorectal cancer with conventional laparoscopic surgery (CLS).

**Methods:**

Between June 28, 2017, and June 29, 2019, a single-center, open-label, non-inferiority, randomized clinical trial was conducted at the Department of General Surgery, Ruijin Hospital (North), Shanghai Jiaotong University School of Medicine in Shanghai, China. In total, 200 patients diagnosed or suspected of colorectal cancer (cT_1–4a_N_0–2_M_0_) were randomly assigned to either the SILS or CLS group in a 1:1 ratio. The primary outcome was early morbidity rate. Secondary outcomes included intraoperative outcomes, pain intensity, postoperative recovery, pathologic outcomes, and long-term outcomes.

**Results:**

In total, 193 participants (SILS, 97; CLS, 96) were analyzed in the modified intention-to-treat (MITT) population. Among them, 48 underwent right hemicolectomy (SILS *n* = 23, 23.7% and MLS *n* = 25, 26%), 15 underwent left hemicolectomy (SILS *n* = 6, 6.2% and MLS *n* = 9, 9.4%), 1 underwent transverse colectomy (MLS *n* = 1, 1%), 57 underwent sigmoidectomy (SILS *n* = 32, 33% and MLS *n* = 25, 26%), and 72 underwent anterior resection (SILS *n* = 36, 37.1% and MLS *n* = 36, 37.5%). No significant differences were observed in the baseline characteristics. The intraoperative complication was comparable between the two groups [5 (5.2%) vs. 4 (4.2%); difference, 1%; 95% CI, −5.8% to 7.8%; *p* > 0.999) and so was postoperative complication rates [10 (10.3%) vs. 14 (14.6%); difference, −4.3%; 95% CI, −13.9% to 5.3%; *p* = 0.392]. The SILS group showed shorter incision length [median (IQR), 4 (3.5–5) vs. 6.6 (6–7.5), *p* < 0.001] and lower VAS scores on the first [median (IQR), 4 (3–5) vs. 4 (4–5), *p* = 0.002] and the second day [median (IQR), 2 (1.5–3) vs. 3 (2–4), *p* < 0.001] after surgery. No statistically significant difference was found in other measured outcomes.

**Conclusions:**

Compared with CLS, SILS performed by experienced surgeons for selected colorectal cancer patients is non-inferior with good short-term safety and has the advantage of reducing postoperative pain.

**Clinical Trial Registration:**

ClinicalTrials.gov, identifier NCT03151733.

## Introduction

At present, surgery is among the most important treatments for colorectal cancer. Laparoscopic surgery is becoming a major option since several randomized controlled trials (1–6) have demonstrated its safety, effectiveness, and benefits in less intraoperative blood loss, faster recovery, less postoperative pain, shorter hospital stays, etc. compared with laparotomy. With the continuous development of minimally invasive technology and instruments, more and more studies focus on further reducing surgical trauma. Single-incision laparoscopic surgery (SILS) is attracting increasingly more attention as an attempt to transition to “scarless” surgery. It has been a decade since Bucher et al. (7) first reported SILS for colon cancer, and this technique is considered to be the next major advance in the evolution of minimally invasive surgical approaches to colorectal disease feasible in generalized use (8). However, to date, the technique is still in its early stage and is controversial, especially regarding its technical challenges, safety in rectal cancer, potential benefits of reducing postoperative pain and better cosmetic effects, and long-term oncological outcomes (9–11). The evidence is too sparse to allow any firm recommendation. Therefore, more studies, especially large-scale, prospective, randomized controlled trials are needed to further evaluate its application in colorectal cancer. Our center first performed SILS for colorectal cancer in December 2013 and found it to be a safe and feasible option, which inspired us to conduct this RCT to test the hypothesis. Patients are still being followed up and the short-term outcomes of the study are presented here.

## Materials and Methods

### Design

This single-center, open-label, prospective, randomized, non-inferiority trial (ClinicalTrials.gov identifier: NCT03151733) was conducted at the Department of General Surgery, Ruijin Hospital (North), Shanghai Jiaotong University School of Medicine in Shanghai, China. The study protocol and the informed consent documents were approved by the Clinical Trial Ethics Committee of Ruijin Hospital (North).

### Participants

Patients aged 18 to 85, diagnosed with or suspected of colorectal cancer with clinical stage of cT_1–4a_N_0–2_M_0_, were screened for inclusion. Considering the controversy of laparoscopic surgery for lower rectal cancer and the SILS technical difficulties, patients with body mass index (BMI) >30 kg/m (2), tumor size >5 cm, gastrointestinal surgery history (apart from appendicectomy), or tumor lower border located distal to the peritoneal reflection were excluded. The detailed inclusion, exclusion, and withdrawal criteria are shown in **Table 1**. Written informed consents were received from all participants.

**Table 1:** Inclusion, exclusion, and withdrawal criteria.

Inclusion criteria	Exclusion criteria	Withdrawal criteria
• 18 years < age < 85 years	• BMI > 30 kg/m (2)	• Intraoperative or pathological confirmation of invasion of adjacent structures or distant metastasis
• Pathological or highly suspected colorectal carcinoma	• The lower border of the tumor is located distal to the peritoneal reflection	• Non-colorectal adenocarcinoma confirmed by postoperative pathology
• Tumor located in the colon and rectum (the lower border of the tumor is above the peritoneal reflection)	• Previous gastrointestinal surgery (apart from appendicectomy)	• Requirement of emergency operation due to the change of illness state
• Clinically diagnosed cT_1–4a_N_0–2_M_0_ lesions according to the seventh Edition of AJCC Cancer Staging Manual	• Emergency operation due to complication caused by colorectal cancer (bleeding, perforation, or obstruction)	• Inability to undergo surgery or anesthesia due to the change of illness state
• Tumor size of ≤5 cm	• Requirement of simultaneous surgery for other disease	• Unable to complete the clinical trial due to various reasons
• Performance status ECOG 0–1	• Pregnancy or lactation	• Patient required to withdraw
• ASA class I to III	• Severe mental disease	
• Informed consent	• Simultaneous or metachronous multiple cancers with disease-free survival ≤5 years	

BMI, body mass index; ASA, The American Society of Anesthesiologists; ECOG, Eastern Cooperative Oncology Group.

### Randomization and Masking

Eligible patients were randomly assigned to either the SILS or conventional laparoscopic surgery (CLS) group in a 1:1 ratio. The data inspector, who did not participate in patient screening and enrollment, performed the randomization using the random number table method. The allocation sequence was concealed from the surgeons until participants were formally assigned to their groups, using sequentially numbered, identical, opaque, sealed envelopes. Operative procedures and postoperative treatment were not concealed from the patients or investigators.

### Surgical Procedures

Six qualified surgeons with over 50 cases of experience of laparoscopic colorectal cancer surgery performed the operations in the CLS group, while the SILS group operations were all performed by the same surgeon (RZ), who had performed over 100 cases of SILS for colorectal cancer before the trial began.

After general anesthesia, the patients were placed in optimal positions according to the surgical approach. In general, straddle-type supine, Trendelenberg with left-tilted or right-titled position was used in right colectomy or left colectomy, respectively, and modified lithotomy, Trendelenberg, right-tilted position was used in sigmoidectomy and anterior resection.

In the SILS group, a SILS™ Port (Covidien, Mansfield, MA, USA) with three 5-mm cannulas inserted or a Star-Port (Surgaid^®^, Guangzhou, China) consisting of three fixed instrument channels (one 5 mm, two 10 mm, and one 12 mm) was installed through a 2~3 cm in length midline periumbilical incision. A 30° laparoscope, a 0° flexible laparoscope (LTF-VP, Olympus Medical Systems, Tokyo, Japan), or an Olympus 3D laparoscope was used based on the choice of port. In cases using the SILS™ Port, the main operating cannula was changed from 5 to 12 mm when using Endo GIA™. In the CLS group, the operation was performed with 3 to 5 trocars including a 12-mm trocar for a 30° laparoscope or a 3D laparoscope in the periumbilical area. The main operating trocar was 12 mm, while the remaining trocars were 5 mm. All operations in both groups were performed using conventional laparoscopic instruments.

All the operations were performed according to the same oncologic principles, including complete mesocolic excision (CME) for colon cancer and total mesorectal excision (TME) for rectal cancer with D3 lymph node dissection. The medial-to-lateral or lateral-to-medial approach was at the discretion of the surgeon. For sigmoidectomy and anterior resection, mobilization of the splenic flexure was not performed routinely except in cases of a lack of redundancy of the sigmoid colon or excessive anastomotic tension. Depending on the anastomosis, the prophylactic ileostomy may be performed.

The specimen was retrieved through the wound protector installed through the transumbilical incision (SILS group) or a 3- to 4-cm additional incision (CLS group). The draining tube was extracted through the incision in the SILS group or through the main operating channel in the CLS group. The closure of incisions was done by absorbable monofilament. The details of the operative procedure were described in our previous reports (12, 13).

### Perioperative Management

The perioperative management was similar between the two groups. All the patients underwent mechanical bowel preparation and oral antibiotic prophylaxis 1 day before surgery. The Foley catheters for patients who underwent anterior resections were removed after bladder training by clamping, while others were removed on postoperative day 1. Pain was controlled exclusively within 48 h after operation by patient-controlled analgesia (PCA, 100 ml) composed of 2 μ g/kg sufentanil citrate and 100 mg flurbiprofen axetil. The PCA continued to infuse at 2 ml/h. If the pain could not be tolerated, the patient could receive a bolus dose of 2 ml, with a locking time of 20 min between the doses. Additional analgesics were allowed in cases of breakthrough pain. Patients were allowed to drink water after first passage of flatus and then gradually transitioned to a liquid and soft diet. The drainage tube was removed 1~2 days after restoration of soft diet. Discharge was considered when the following conditions were met: no fever or other signs of complications and tolerating soft diet and controlled pain without any analgesics.

### Outcomes

The primary outcome was early morbidity defined as the postoperative complications observed within 30 days after surgery. It was graded according to the Clavien–Dindo classification. The secondary outcomes included intraoperative outcomes (operation time, estimated blood loss, incision length, conversion rate), postoperative pain score, postoperative recovery (time to first ambulation, flatus, liquid diet and soft diet, length of hospital stay), pathologic outcomes (tumor size, number of harvested lymph nodes, proximal and distal resection margins), and long-term outcomes (5-year incision hernia rate, 3-year disease free survival rate, 5-year overall survival rate). The incision length was defined as the sum of all incision lengths. Postoperative pain was recorded using the visual analog scale (VAS) pain score (0–10 points) on postoperative days 1, 2, and 3. The pathologic outcomes were evaluated by pathologists. The follow-up was consistent with the National Comprehensive Cancer Network (NCCN) guidelines. Recurrence was confirmed by radiological or histological methods.

### Sample Size Estimation

Sample size estimation was performed with PASS (11th edition, NCSS, LLC, UT, USA). According to previous data of our center, primary endpoint (early morbidity rate) was estimated to be 14% and 10%, respectively, in the CLS group and SILS group. The sample size was determined with one-side alpha of 0.025, a power of 0.8, and a non-inferiority margin of 10%. Assuming a dropout rate of 15%~20%, the sample size was estimated as 200 (100 per group).

### Statistical Analysis

Statistical analysis was performed with SPSS (version 22.0, SPSS Inc., Chicago, IL, USA). Continuous data were described as means with standard deviations (SD) or median with interquartile ranges (IQR), and categorical data were described as frequencies and percentages. Statistically significant differences were evaluated using the Mann–Whitney *U* test, Student’s *t*-test, *χ^2^* test, and Fisher’s exact test, as appropriate. A two-sided *p*-value <0.05 was considered statistically significant. Data were analyzed in the modified intention-to-treat (MITT) population.

## Results

**Figure 1:**
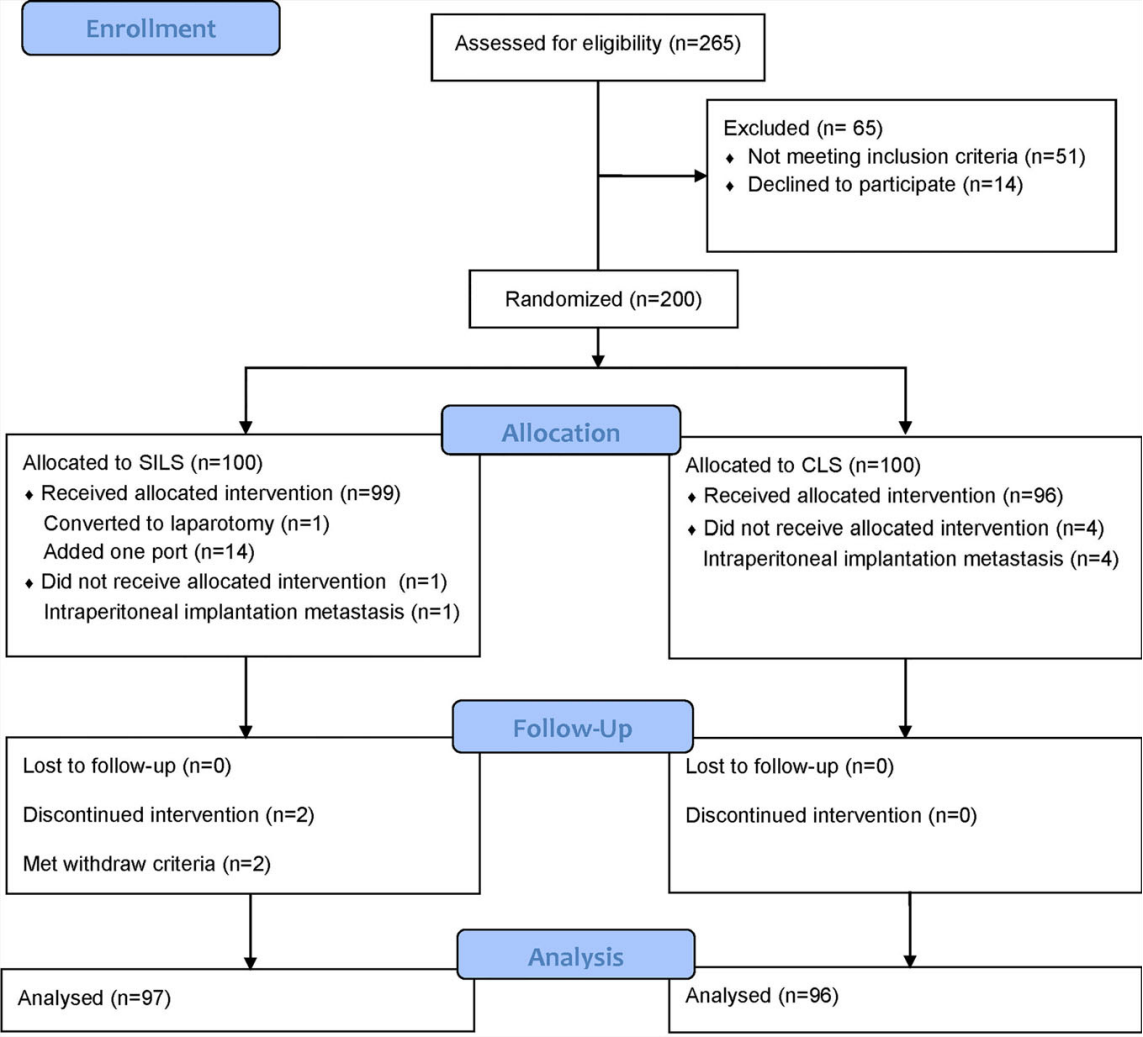
Consort flow diagram.

Between June 28, 2017, and June 29, 2019, 200 patients were randomly assigned to either the SILS or CLS group. A total of 193 patients [SILS: 97, of whom 56 were male (57.7%), with a median (IQR) age of 63 (54.5–69) years; CLS: 96, of whom 54 were male (56.3%), with a median (IQR) age of 65 (56–70) years] were analyzed in the MITT population (**Figure 1**). Forty-nine right hemicolectomy (SILS: 23, 23.7% and CLS: 26, 27.1%), 15 left hemicolectomy (SILS: 6, 6.2% and CLS 9, 9.4%), 1 transverse colectomy (CLS: 1, 1%), 55 sigmoidectomy (SILS: 31, 32% and CLS: 24, 25%), and 73 anterior resection (SILS: 37, 38.1% and CLS: 36, 37.5%) were performed. The baseline characteristics were well balanced between the groups (**Table 2**).

**Table 2:** Baseline characteristics.

Characteristics	SILS (*n* = 97)	CLS (*n* = 96)
Age (years)^a^	63 (54.5–69)	65 (56–70)
Sex ratio (M:F)	56:41	54:42
BMI (kg/m^2^)^b^	23.0 (2.8)	23.6 (3.2)
ASA grade		
I	40 (41.2)	29 (30.2)
II	47 (48.5)	53 (55.2)
III	10 (10.3)	14 (14.6)
Comorbidities	51 (52.6)	53 (55.2)
Previous abdominal surgery	23 (23.7)	26 (27.1)
ECOG score		
0	43 (44.3)	36 (37.5)
1	54 (55.7)	60 (62.5)
Procedure performed		
Right hemicolectomy	23 (23.7)	26 (27.1)
Left hemicolectomy	6 (6.2)	9 (9.4)
Transverse colectomy	0 (0)	1 (1)
Sigmoidectomy	31 (32.0)	24 (25.0)
Anterior resection	37 (38.1)	36 (37.5)

Values in parentheses are percentages unless indicated otherwise.

BMI, body mass index; ASA, The American Society of Anesthesiologists; ECOG, Eastern Cooperative Oncology Group.

a Values are mean (SD).

b Values are median (IQR).

### Intraoperative and Postoperative Outcomes

The intraoperative and postoperative outcomes are shown in **Table 3**. The median (IQR) operation time was similar between the groups [120 (90–132) vs. 120 (96.3–148.3) min, *p* = 0.262]. No conversion occurred in the CLS group, while 14 patients (14.4%) used additional trocars (all cases plus one trocar) and 1 patient (1%) converted to laparotomy in the SILS group. The reasons for additional trocars were intraperitoneal adhesion (*n* = 3, 3.1%), vascular injury (*n* = 4, 4.1%), adjacent organ injury (*n* = 1, 1%), poor surgical exposure (*n* = 4, 4.1%), and dissection difficulties (*n* = 2, 2.1%). The reason for laparotomy was dissection difficulties. The median (IQR) incision length was significantly shorter in the SILS group [4 (3.5–5) vs. 6.6 (6–7.5) cm, *p* < 0.001]. The median (IQR) VAS scores in the SILS group were lower on postoperative day 1 (POD1) [4 (3–5) vs. 4 (4–5), *p* = 0.002] and POD2 [2 (1.5–3) vs. 3 (2–4), *p* < 0.001]. The usage of additional postoperative analgesics within 3 days after surgery was comparable between the two groups [13 (13.4%) vs. 11 (11.5%), *p* = 0.828]. The estimated blood loss and recovery from surgery did not differ statistically between the two groups.

**Table 3:** Intraoperative and postoperative outcomes.

Variable	SILS (*n* = 97)	CLS (*n* = 96)	*P* ^a^
Operation time (min)^b^	120 (90–132)	120 (96.3–148.3)	0.262^c^
Estimated blood loss (ml)^b^	50 (10–100)	50 (20–100)	0.067^c^
Conversions	15 (15.5)	0 (0)	
Laparotomy	1 (1)	0 (0)	>0.999
Additional trocar	14 (14.4)	–	
Incision length (cm)^b^	4 (3.5–5)	6.6 (6–7.5)	<0.001^c^
Time to first ambulation (h)^b^	48 (24–48)	48 (48–72)	0.054^c^
Time to flatus (h)^b^	48 (46.5–72)	48 (48–72)	0.341^c^
Time to liquid diet (days)^b^	5 (5–6)	5 (5–6)	0.501^c^
Time to soft diet (days)^b^	7 (6–7)	7 (6–7.8)	0.763^c^
Length of hospital stay (days)^b^	8 (8–10)	8 (8–10)	0.613^c^
Postoperative pain score (VAS)^b^			
POD1	4 (3–5)	4 (4–5)	0.002^c^
POD2	2 (1.5–3)	3 (2–4)	<0.001^c^
POD3	1 (1–2)	1.5 (1–2)	0.316^c^
Additional postoperative analgesics	13 (13.4)	11 (11.5)	0.828
POD1	10 (10.3)	5 (5.2)	0.282
POD2	6 (6.2)	8 (8.3)	0.592
POD3	3 (3.1)	3 (3.1)	>0.999
Intraoperative complications	5 (5.2)	4 (4.2)	>0.999
Vascular injury	4 (4.1)	4 (4.2)	
Adjacent organ injury	1 (1)	0 (0)	
Postoperative complications	10 (10.3)	14 (14.6)	0.392
Peritoneal effusion	2 (2.1)	2 (2.1)	
Anastomotic leakage	3 (3.1)	1 (1)	
Wound infection	4 (4.1)	2 (2.1)	
Anastomotic hemorrhage	0 (0)	1 (1)	
Intra-abdominal hemorrhage	0 (0)	1 (1)	
Ileus	0 (0)	2 (2.1)	
Urinary retention	0 (0)	2 (2.1)	
CVC infection	0 (0)	1 (1)	
FUO	1 (1)	2 (2.1)	
Grade of complications			0.669
I	7 (7.2)	9 (9.4)	
II	1 (1)	3 (3.1)	
IIIa	0 (0)	1 (1)	
IIIb	2 (2.1)	1 (1)	
Reoperation	2 (2.1)	1 (1)	>0.999
Readmission within 30 days of surgery	1 (1)	0 (0)	>0.999
Mortality within 30 days of surgery	0 (0)	0 (0)	–

Values in parentheses are percentages unless indicated otherwise.

VAS, visual analog score; POD, postoperative day; CVC, central venous catheters; FUO, fever of unknown origin.

a χ^2^ or Fisher’s exact test.

b Values are median (IQR).

c Mann–Whitney U test.

The intraoperative complication [5 (5.2%) vs. 4 (4.2%); difference, 1%; 95% CI, −5.8% to 7.8%; *p* > 0.999] and postoperative complication rates [10 (10.3%) vs. 14 (14.6%), *p* = 0.392] were comparable between the two groups. Non-inferiority of SILS compared with CLS demonstrated as the upper limit of the 95% confidence interval (CI) for between-group difference calculated by the Newcombe method was less than the non-inferiority margin of 10% (postoperative complication rate difference, −4.3%; 95% CI, −13.9% to 5.3%). One patient (1%) in the SILS group had splenic injury during left hemicolectomy. The other eight patients (SILS: 4, 4.1%; CLS: 4, 4.2%) were vascular injury. The postoperative complications of the SILS group included two (2.1%) peritoneal effusion (grade I), three (3.1%) anastomotic leakages (grade IIIa: 1, 1%; grade IIIb: 2, 2.1%), four (4.1%) wound infections (grade I), and one (1%) fever of unknown origin (FUO, grade I). The postoperative complications of the CLS group included two (2.1%) peritoneal effusion (grade I), one (1%) anastomotic leakage (grade IIIb: 1), two (2.1%) wound infections (grade I), one (1%) anastomotic hemorrhage (grade I), one (1%) intra-abdominal hemorrhage (grade II), two (2.1%) ileus (grade II), one (1%) urinary retention (grade IIIa), one (1%) central venous catheter infection (grade I), and one (1%) FUO (grade I). Two patients (SILS: 1, 1%; CLS: 1, 1%) performed emergency reoperation with diverting ileostomy during the hospitalization. One patient (1%) in the SILS group was readmitted for anastomotic leakage 2 days after discharge and performed diverting ileostomy. There was no mortality within 30 days after surgery in either group.

### Pathologic Outcomes

Regarding the pathologic outcomes, the tumor size, proximal and distal resection margins, number of harvested lymph nodes, cell type, neurovascular invasion, and pathologic stage were similar between the two groups (**Table 4**). In the cases of rectal cancer, no positive circumferential resection margin was found.

**Table 4:** Pathologic outcomes.

Variable	SILS (*n* = 97)	CLS (*n* = 96)	*P* ^a^
Tumor size (cm)^b^	3.5 (2.5–4)	4 (3–4.5)	0.071^c^
Proximal resection margins (cm)^b^	6 (4–9)	6 (4.1–9.9)	0.422^c^
Distal resection margins (cm)^b^	4 (2.8–7)	4.4 (2.5–7.9)	0.527^c^
Harvested lymph nodes^b^	13 (10–15)	13 (10.2–15)	0.952^c^
Cell type			0.195
WD/MD	50 (51.5)	40 (41.7)	
PD/others	47 (48.5)	56 (58.3)	
Perineural invasion	21 (21.6)	18 (18.8)	0.720
Vascular invasion	32 (33.0)	25 (26)	0.344
Positive circumferential resection margin^d^	0 (0)	0 (0)	–
pT stage			0.504
Tis/T1	18 (18.6)	12 (12.5)	
T2	20 (20.6)	20 (20.8)	
T3	32 (33.0)	29 (30.2)	
T4a	27 (27.8)	35 (36.5)	
pN stage			0.619
N0	61 (62.9)	65 (67.7)	
N1	28 (28.9)	22 (22.9)	
N1a	11 (11.3)	10 (10.4)	
N1b	13 (13.4)	10 (10.4)	
N1c	4 (4.1)	2 (2.1)	
N2	8 (8.2)	9 (9.4)	
N2a	6 (6.2)	8 (8.3)	
N2b	2 (2.1)	1 (1)	
pTNM stage			0.671
0	4 (4.1)	5 (5.2)	
I	28 (28.9)	24 (25.0)	
II	29 (29.9)	36 (37.5)	
IIA	17 (17.5)	18 (18.8)	
IIB	12 (12.4)	18 (18.8)	
III	36 (37.1)	31 (32.3)	
IIIA	6 (6.2)	1 (1)	
IIIB	25 (25.8)	26 (27.1)	
IIIC	5 (5.2)	4 (4.2)	

Values in parentheses are percentages unless indicated otherwise.

WD, well differentiated; MD, moderately differentiated; PD, poorly differentiated.

a χ^2^ or Fisher’s exact test.

b Values are median (IQR).

c Mann–Whitney U test.

d Assessed in rectal cancer.

## Discussion

To the best of our knowledge, seven RCT studies (9, 11, 14–19) have been published on SILS for colorectal cancer, including three multicenter studies (9, 11, 18, 19). However, these studies all had limitations. The conclusions of four previous RCT studies (14–17) may be less reliable because of the inadequate sample size calculations. Besides, in the multicenter study of Maggiori et al. (19), the application value of SILS for colorectal cancer could not be well evaluated since both benign and malignant cases were included and the sample size of malignant cases was small (18 patients each group). The SIMPLE trial (9) was the best designed RCT to date, with the largest sample size and multicenter participation. However, similar to an earlier multicenter RCT study in Japan (11, 18), it excluded patients with rectal, descending colon, and transverse colon cancers. Thus, we try to overcome such limitation and add more evidence to the literature while conducting the present study.

In the present study, the non-inferiority was met and the early morbidity was comparable between the SILS and CLS groups. In addition, the conversion rate of SILS (15.5%) was similar to previous RCT studies (9, 16–18) and the operation time did not increase, suggesting that SILS for selected patients performed by an experienced surgeon is short-term safe and feasible.

The SILS group showed lower VAS scores on POD1 and POD2 with similar postoperative analgesics usage in the present study, which may be related to fewer incisions. However, the recovery process in the SILS group did not speed up compared with the CLS group. Patient management greatly affects the postoperative recovery process. In the study of Osborne et al. (20), the patients received enhanced recovery after surgery (ERAS), and the postoperative hospital stay in the SILS group for high anterior resection was 1 day, which was faster than that in the CLS group (3 days).

Total incision length is commonly used to evaluate cosmetic effects. As reported above, the SILS group had a shorter incision length because of fewer trocars. However, cosmetic effect is a subjective feeling not only determined by the incision length. Some reported scales and questionnaires may be more suitable for the evaluation of cosmetic effect (21, 22). In addition, the informed consent process was very important for the description of the surgical scar site and size, which would directly affect the psychological recognition and acceptance of the incision (23).

In terms of long-term outcomes, only one RCT study (11) and a few retrospective studies (24–26) have been reported. These studies showed comparable 3- or 5-year survival rates in both groups. In addition, long-term follow-up is needed to determine whether SILS increases the incidence of incisional hernia. In the present trial, all patients are currently being followed up, and long-term outcomes will be reported when all study endpoints have been reached.

The inclusion and exclusion criteria of the current study were strict due to technical challenges associated with SILS. Indications of SILS for colorectal cancer still need to be explored. In the study of Jung et al. (27), among the 144 cases of LAR and 3 cases of APR, one additional trocar was needed in 107 cases because of the special complexity and difficulty on distal division with insufficient angled stapler and proper total mesorectal excision, which was considered as a second-string procedure. We argue that this result suggests that SILS may not be appropriate for rectal cancer with low tumor sites, and hence, these patients were excluded from the study.

At present, the development of SILS for colorectal cancer is mainly limited by the technical challenges, including loss of triangulation, parallel coaxial effect, poor exposure, and instrument collision. The unique skill sets cannot be directly adapted from existing conventional laparoscopic surgery experience (28). The internal instrument cross and external hand cross technique are the main methods to restore the triangulation. The introduction of 3D laparoscope and flexible laparoscope, reasonable position adjustment, and suspension technique can effectively expose the surgical field. In the future, with the integration of instrument functions and the application of robotic surgery, the difficulty of SILS will be hopefully further reduced.

This study has several limitations. First, the inclusion and exclusion criteria were very strict, especially that the exclusion criteria of BMI >30 kg/m^2^ would exclude a large number of patients in more industrialized countries. Second, the SILS were all performed by the same senior surgeon, limiting the generalizability of the findings. Third, the protocol did not include ERAS protocols which have become routine at most institutions, so the assessment of postoperative recovery may not be entirely reliable.

## Conclusions

Compared with CLS, SILS performed by experienced surgeons for selected colorectal cancer patients is non-inferior with good short-term safety and has the advantage of reducing postoperative pain.

## Data Availability Statement

The raw data supporting the conclusions of this article will be made available by the authors, without undue reservation.

## Ethics Statement

The studies involving human participants were reviewed and approved by the Clinical Trial Ethics Committee of Ruijin Hospital (North). The patients/participants provided their written informed consent to participate in this study.

## Author Contributions

RZ, XJ, and TZ had full access to all of the data in the study and take responsibility for the integrity of the data and the accuracy of the data analysis. Concept and design: RZ, ZS, KL, XJ, and TZ. Acquisition, analysis, or interpretation of data: all authors. Drafting of the manuscript: ZS, KL, YL, XJ, TZ, and RZ. Critical revision of the manuscript for important intellectual content: ZS, KL, YL, XJ, TZ, and RZ. Statistical analysis: ZS, KL, and YS. Obtained funding: RZ. Administrative, technical, or material support: all authors. Supervision: RZ, XJ, and TZ. All authors contributed to the article and approved the submitted version.

## Funding

This study was supported by the Advanced and Appropriate Technology Promotion Projects of Shanghai Municipal Health Commission (2019SY058) and Clinical Skills and Innovations 3-Year Program of Shanghai Hospital Development Center (SHDC2020CR1026B).

## Conflict of Interest

The authors declare that the research was conducted in the absence of any commercial or financial relationships that could be construed as a potential conflict of interest.

## Publisher’s Note

All claims expressed in this article are solely those of the authors and do not necessarily represent those of their affiliated organizations, or those of the publisher, the editors and the reviewers. Any product that may be evaluated in this article, or claim that may be made by its manufacturer, is not guaranteed or endorsed by the publisher.
